# JMIR Dermatology’s 2023 Year in Review

**DOI:** 10.2196/57007

**Published:** 2024-09-17

**Authors:** Ramiro Rodriguez, Robert P Dellavalle

**Affiliations:** 1 Department of Dermatology University of Colorado School of Medicine Aurora, CO United States; 2 Department of Dermatology Minneapolis VA Medical Center US Department of Veterans Affairs Minneapolis, MN United States; 3 Department of Dermatology University of Minnesota School of Medicine Minneapolis, MN United States

**Keywords:** rural, teledermatology, neglected tropical diseases, NTD, melanoma, PubMed, review, diversity, editorial, dermatology, review, JMIR

## Abstract

In 2023, *JMIR Dermatology* embraced papers treating all topics related to diseases of the skin, hair, and nails. This editorial aims to bring attention and recognize reviewers, staff, and authors for their contributions to the journal. *JMIR Dermatology* updated the Research Letter format and introduced the In Memorium article type to feature and celebrate highly accomplished and internationally recognized leaders in dermatology. We also summarize the 3 *JMIR Dermatology* publications from 2023 with the highest Altmetric scores and share what we look forward to in the coming year.

In 2023, *JMIR Dermatology* embraced papers treating all topics related to diseases of the skin, hair, and nails. This editorial aims to bring attention to and recognize reviewers, staff, and authors for their contributions to the journal. *JMIR Dermatology* published more than 78 papers exploring clinical information exchange, education, and efforts to facilitate the diagnosis and delivery of dermatologic care. These publications included Original Articles (n=29), Reviews (n=9), Research Letters (n=31), Viewpoints (n=3), a Short Paper (n=1), Case Reports (n=2), Editorials (n=2), and an In Memoriam article (n=1), which have provided room for authors to showcase their research.

Research Letters were a popular article type; this article type is “optimal for presenting new, early, or sometimes preliminary research findings, including interesting observations from ongoing research with significant implications that justify concise and rapid communication” [1]. The Research Letter article type is also conducive to JMIR Publications' cascading peer review policy [2].

The In Memoriam article type was added to feature and celebrate highly accomplished and internationally recognized leaders in dermatology. Dr William Weston was featured in this year’s In Memoriam article to recognize his accomplishments as a clinician and educator and for inspiring the careers of many academic dermatologists [3].

The 3 publications with the highest Altmetric scores (as of December 4, 2023) examined the strengths of teledermatology applications and areas for improving care delivery. Drabarek et al [4] provided qualitative data capturing patient impressions of a participant-led skin self-examination (SSE) model using smartphone app reminders, patient-performed dermoscopy, and teledermatology assessments. Implementation barriers revolved around participants’ perceptions of melanoma recurrence risk. Concerns included the short time interval between melanoma diagnosis, treatment, and the introduction of the SSE model; medical provider availability for urgencies; and the need for evidence on the efficacy of SSE relative to usual care. Follow-up studies could explore underserved communities where access to dermatologic care is a preexisting limitation. Yotsu et al [5] exemplified studies exploring the use of phone apps in rural, underserved communities; they evaluated how teledermatology can diagnose skin-related neglected tropical diseases. Over 3 months in rural Côte d’Ivoire, the investigators trained local practitioners and implemented an app-based electronic medical record and teledermatology workflow. The study design assigned providers to an interventional group and a usual care group. It was found that the group who used the app-based electronic medical record and teledermatology workflow identified 79 cases of different neglected tropical diseases (Buruli ulcer, leprosy, lymphatic filariasis, mycetoma, scabies, and yaws), compared to the 8 identified cases among practitioners not using the system. Difficulties with the system were reported to revolve around internet connectivity and initial challenges with using the system. Challenges with teledermatology were not only limited by internet connectivity but also by target diseases, as described by Long et al [6]. Specifically, teledermatology posed unique challenges for treating hidradenitis suppurativa. These challenges involved the sensitive location of lesions and the limited nature of a teledermatology exam.

Overall, teledermatology offers an opportunity to bring health equity to diverse populations, and *JMIR Dermatology* embraced these topics [7]. With new technological advances, *JMIR Dermatology* looks forward to improving the understanding of teledermatology applications in the coming years. Through the contributions of authors, the editorial team, and reviewers, the Library Operations Division of the US National Library of Medicine decided to include *JMIR Dermatology* for indexing in PubMed Central after a rigorous scientific and technical evaluation. This indexing will be in addition to the journal appearing in the Sherpa Romeo, Scopus, Directory of Open Access Journals, and Centre for Agriculture and Biosciences International databases. These platforms will help increase the visibility of the authors’ work and help advance clinical information exchange and education.

Beyond our 3 highlighted papers, our PubMed indexing has made 2023 a big year for *JMIR Dermatology*. We are proud to be open access and to be the number 1 academic dermatology journal published in Canada per the Scimago Journal & Country Rank. We sincerely thank the International Society of Digital Health in Dermatology, formerly known as The International Teledermatology Society, and all of our editorial board members [8], staff, and reviewers (Multimedia Appendix 1), without whom our success would not have been possible.

## Appendix

Multimedia Appendix 1 *JMIR Dermatology* reviewers in 2023.

## Abbreviations

SSE: skin self-examination
