# Supplementary material for: JMIR Dermatology’s 2023 Year in Review
Source: JMIR Dermatol. 2024 Sep 17;7:e57007. doi: 10.2196/57007 (PMC11445624; doi:10.2196/57007)
Supplement: Multimedia Appendix 1 [file derma_v7i1e57007_app1.docx]

**Multimedia Appendix 1.** JMIR Dermatology reviewers in 2023.

| Last Name | First Name | Last Name | First Name |
| --- | --- | --- | --- |
| Kumar | Siva | Bernardes-Souza | Breno |
| Abdalla | Mohamed | Beyer | Fiona |
| Acquaviva | Kimberly | Bhadra | Presenjit |
| Adelman | Madeline | Bhatt | Harshil |
| Afzal | Sina | Bidmon | Sonja |
| Aggarwal | Pushkar | Bie | Bijie |
| Aktaş | Habibullah | Bjerkan | Jorunn |
| AL-Asadi | Ali | Black | Austin |
| Alhanshali | Lina | Bock | Beth |
| Alhusayen | Raed | Boumans | Roel |
| Alkhalifah | Azzam | Bowen | Deborah |
| Allam | Ayman | Bowers | Sacharitha |
| Allem | Jon-Patrick | Brahmbhatt | Viraj |
| Al-Rawi | Ahmed | Brenaut | Emilie |
| Alsalhi | Hamza | Brooke | John |
| Amon | Krestina | Brooks | Ian |
| Anaje | Chetanna | Bu | Yi |
| Anand | Sahil | Buller | David |
| Anastasiou | Athanasios | Buller | Mary |
| Andrikopoulou | Elisavet | Burnette | Colin |
| Aqajari | Seyed Amir Hossein | Cacciamani | Giovanni |
| Arbabisarjou | Azizollah | Camillo | Cheryl |
| Arents | Bernd | Cao | Annie |
| Arnet | Isabelle | Carrion | Carme |
| Ashack | Kurt | Carvalho | Darlinton |
| Ashraf | Shahzad | Ceron | Wilson |
| Aslanidis | Theodoros | Chang | Yu-Feng |
| Avis | Jillian | chaudhry | sharjeel |
| Ayatollahi | Haleh | Chen | Qingwei |
| Azadnajafabad | Sina | Cheng | Xi |
| Bacha | Aziz-Ur-Rahim | Cheudjeu | Antony |
| Bailey | Adrian | Chitranshi | Anany |
| Balzer | Felix | Chiu | Hung-Wen |
| Bao | Bokan | Chong | Kimberly |
| Baradaran | Hamidreza | Chowdhary | Paraag |
| Barbosa | Maria | Chrimes | Dillon |
| Barsoum | Christina | Chu | Brian |
| Barwick | Melanie | Chu | Yuanchia |
| Basch | Corey | Chung | Jamie |
| Batrakoulis | Alexis | Chung | Siubak |
| Baumrin | Emily | Ciccarese | Giulia |
| Bautista | John Robert | Claggett | Jennifer |
| Behzadifar | Masoud | Collier | Sigrid |
| Benis | Arriel | Coumoundouros | Chelsea |
| Benítez-Andrades | José Alberto | Curcio | Natalie |
| Benson | Heather | da Silva e Souza Miranda | Pedro Augusto |
| Berardesca | Enzo | Davies | William |
| Davison | Kelly | Gasmi | Maha |
| De Decker | Ignace | George | Immanuel Victor |
| De Graeve | Diane | Geronemus | Roy G |
| De Vita | Valerio | Gessain | Antoine |
| Dessinioti | Clio | Gibson | Candace |
| Devaraj | Dinesh | Giraud-Carrier | Christophe |
| Dexter | Franklin | Gissel | Christian |
| Di Stasio | Dario | Glanz | Karen |
| Dicker | Tony | Gokdemir | Ozden |
| Do | Huyen Phuc | Gomez | Francisco |
| do Vale de Souza | Isabella | Gorman | Shelley |
| Doan | Son | Gray | Hope |
| Domnich | Alexander | Gu | Alex |
| Dong | Tianyu | Guardabasso | Vincenzo |
| Dotson | W. David | Guevara | Bryan Edgar |
| Downie | Simone | Gulliver | Susanne |
| Drago | Francesco | Gunasekeran | Dinesh |
| Dreno | Brigitte | Guo | Hui |
| Dronyuk | Ivanna | Guo | William |
| Drucker | Aaron | Gupta | Subhas |
| Dunnsiri | Teevit | Hacker | Elke |
| Eapen | Bell | Halperin | Edward |
| Edwards | LaVar | Hamnvik | Ole-Petter |
| El Tantawi | Maha | Hamp | Austin |
| Ellis | Charles | Harada | Yukinori |
| Elpern | David | Harcourt | Diana |
| Elsanousi | Yasir | Hardikar | Navneetha |
| Eswaradass | Prasanna Venkatesan | Hardwicke | Tom |
| Esworthy | Steve | Harpel | Tammy |
| Ewais | Tatjana | Haugsten | Elisabeth Rygvold |
| Fabbrocini | Gabriella | Hayn | Dieter |
| Fang | Wei-Chieh | He | Huan |
| Farid | Ghulam | Heckman | Carolyn |
| Farista | Arshi | Henrique | Nascimento |
| Farzi | Jebraeil | Herman | Ira |
| Feldman | Steven | Hernández-Rodríguez | Juan-Carlos |
| Feldman | Steven | Hertling | Stefan |
| Finny | Abraham | Heslin | Kelly |
| Finstad | Alexandra | Hidig | Sakarie |
| Fioratou | Evridiki | Hidki | Asmaa |
| Fölster-Holst | Regina | Hofford | Mackenzie |
| Fonseka | Lakshan | Hook Sobotka | Michelle |
| Forni | Cristiana | Hopkins | Zach |
| Friedman | Sara | Horsham | Caitlin |
| Fultz Hollis | Kate | Hosseini | Mohammad-Salar |
| Gabashvili | Irene | Hu | Dian |
| Gamieldien | Yasin | Huang | Austin |
| Ganesh | Shankar | Huang | Ching-Hsin |
| Gao | Xing-Hua | Huang | Victor |
| Ianos | Ana Claudia | Lei | Jianbo |
| Ibrahim | Mohammad | Lepping | Peter |
| Idris | Iszaid | LEUNG | Yuen Ling |
| Iglesias | Lia | Li | Grant |
| Janda | Monika | Li | Yang |
| Jantschi | Lorentz | Li | Zhongqiang |
| Javidnia | Javad | Liebram | Claudia |
| Jenkinson | Jodie | Lin | Cheng-Yu |
| Jensen | Mikkel Bak | Lin | Zhen |
| Jiang | Simon | Lindström | Nataliya |
| Jin | Lei | Lipoff | Jules |
| Johnson | Timothy | Lokker | Cynthia |
| K. | Mikołaj | Long | Valencia |
| Kacem | Imane | Longacre | Meghan |
| Kaczmarczyk | Robert | Longo | Caterina |
| Kaliyadan | Feroze | Lopez Segui | Francesc |
| Kamel | Kevin | LoSicco | Kristen |
| Kanike | Uday | Lotto | Matheus |
| Kapsetaki | Marianna | Lu | J. |
| Kardes | Sinan | Ma | Junjie |
| Kasapcopur | Ozgur | Madhusudhan | Divya |
| Khaw | Wan-Fei | Maghfour | Jalal |
| Khoong | Elaine | Magin | Parker |
| Khordastan | Firoozeh | Mahler | Heike |
| Kian Liang | Goh | Mahmic Kaknjo | Mersiha |
| Kim | Sujin | Mahmoud | Randa Salah |
| Kluczyk | Alicja | Majurul Ahsan | MD |
| Kluger | Nicolas | Makin | Jen |
| Kokolakis | Georgios | Marchetti | Michael |
| koritala | Thoyaja | Marengo | Davide |
| Kotha | Vikas | Marroquin | Nathaniel |
| Krieghoff-Henning | Eva | Mars | Maurice |
| KT | Ashique | Marshall | Robert |
| Kumar | Pavan | Martin | Heather |
| Kunonga | Patience | Matin | Rubeta |
| Kuo | Kuang-Ming | Matthews | Paul |
| Kuru | Timur H. | Mauco | Kabelo Leonard |
| Kvedar | Joseph | Mavragani | Amaryllis |
| Lam | Nikki | Mavrot | Céline |
| Lamy | Francois | Maymone | Mayra |
| Lander | Jonas | McGar | Ashley |
| Lavorgna | Luigi | McMahon | Hayley |
| Law | Terence | Mehdizadeh | Hamed |
| Layton | Alison | Mendes | David |
| Leal Neto | Onicio | Mengel | Eugen |
| Lebwohl | Mark | Miao | Yu |
| Lecomte | Fabienne | Mihalache | Andrew |
| Lee | Sharon | Mikami | Taro |
| Lee | TsungChun | Minichiello | Victor |
| Mircheva | Iskra | Peethambaran | Bejoy |
| Misery | Laurent | Pesälä | Samuli |
| Mohammad Gholi Mezerji | Naser | Pessoa | Débora |
| Mohammed | Heba | Pfeffer | Ulrich |
| Mondal | Himel | Portugal | Crisanta |
| Morgan | Tamara | Prajapati | Stuti |
| Mostafa | Moussa | Pranic | Shelly |
| Moza | Roma | Preclaro | Ivan Arni |
| Mpofu | Rephaim | Price | Carrie |
| Muhammad | Faisal | PS | Shankar |
| Mungoli | Neelesh | Qian | Buyue |
| Muto | Tomoyasu | Qiu | Yan |
| Muzzolon | Mariana | Quan | Theodore |
| Mytle | Dr. Nutan | Quinn | Steve |
| Namba | Hideyuki | Rabbani | Unaib |
| Nambudiri | Vinod | Rajendran | Rahul R |
| Naser | Ahmed | Ramaprasad | Arkalgud |
| Navarro-Triviño | F J | Ramjee | Serena |
| Ndabu | Theophile | Rapelanoro Rabenja | Fahafahantsoa |
| Newman | Jessica | Ray | Keya |
| Nguon | Nina | Ray Chaudhuri | Esha |
| Nguyen | Nhung | Rea | Corinna |
| Nigh | Andrew | Reyes Mugica | Miguel |
| Nikkels | Arjen | Ribeiro | Nuno |
| Niu | Zhaomeng | Ridgers | Nicola |
| Norouzi | Somaye | Riis | Allan |
| Nyman | Elisabeth | Robinson | June |
| Oakley | Amanda | Rodda | Christine |
| Oakley | Amanda M M | Rodriguez | Ramiro |
| O'Connor | Erin | Roguljić | Marija |
| Odutola | Akintola | Ross-White | Amanda |
| Oganesyan | Ani | Rostam Niakan Kalhori | Sharareh |
| Oldenburg | Jan | Roster | Katie |
| Ong | Triton | Routen | Ash |
| op den Buijs | Jorn | Rovetta | Alessandro |
| Ornelas | Jennifer | Rung | Andrea |
| Ortega | Alex | Rutter | Lauren |
| Oska | Sandra | Rytenband | Fernanda |
| Ottwell | Ryan | S | Nickolas |
| Pagoto | Sherry | Sabarguna | Subirosa |
| Pai | Varadraj | Said-Hung | Elias |
| Palmer | Victoria | Salimi | Maryam |
| Pan | Peng | Samuel | Lalitha |
| Pankomera | Richard | Sankaranarayanan | Saiprasad |
| Papadakos | Janet | Santisteban | Antonio |
| Parker | Eva | Sanz-Valero | Javier |
| Patel | Heli | Sapp | Jessica |
| Pathania | Monika | Sarejloo | Shirin |
| Pavliuk | Olena | Schmid-Grendelmeier | Peter |
| Scotts | Sam | Türsen | Ümit |
| Sebastian | Glorin | Umar | Sanusi |
| Shah | Ankur | Umoquit | Muriah |
| Shah | Chintal | van Eenbergen | Mies |
| Shakshouk | Hadir | Van Poucke | Sven |
| Shalaby | Mohammed Nader | Verma | Vipin |
| Shalin | Valerie | Verran | Deborah |
| Shibata | Takakazu | Vestergaard | Tine |
| Shinohara | Michi | Vieira | Rafael |
| Shu | Sara | Vighio | Anum |
| Sibbald | Cathryn | Visser | Adriaan |
| Sideropoulos | Vassilis | Wahbeh | Abdullah |
| Simonart | Thierry | Walkosz | Barb |
| Singh | Gaurav | Wallnöfer | Fabian |
| Singh | Vivek | Wang | Elizabeth |
| Sivesind | Torunn | Wang | Junhui |
| Skidmore | Becky | Wang | Yuli |
| Snoswell | Centaine | Wang | Zhongqing |
| Solomon | James | Warner | Echo |
| Song | Fujian | Watanabe | Chiharu |
| Soofi | Sajid | Weerth | Carsten |
| Sousa-Pinto | Bernardo | Wei | Chapman |
| Southwell | Brian | Wei | Rong |
| Stallings | Elena | Wheless | Lee |
| Steeb | Theresa | Whited | John |
| Stefanov Ketin | Sonja | Whitley | Edgar |
| Steingrimsson | Steinn | Wierzejska | Regina |
| Stobberingh | Ellen E. | Wiesner | Martin |
| Stroh | Jake | Wirth | Felix |
| Su | ShihBin | Wojtara | Magda |
| Suppan | Laurent | Wong | Chiew Meng Johnny |
| Svendsen | Mathias | Wong | Kah Peng |
| Szeto | Mindy | Wright | Marcia |
| Taber | Jennifer | Wu | Hao |
| Tam | Hon Lon | Wu | Huiqun |
| Tang | Chunlei | Wu | Qiwei |
| Tarawneh | Omar | Wyatt | Kirk |
| Taylor | Alan | Xiao | Yi |
| Tensen | E | Xie | Yi |
| Thomas | Jayakar | Xu | Junjie |
| Tibble | Holly | Yan | Chao |
| Tirado-Pérez | José-Pablo | Yang | Chen |
| Tizek | Linda | Yeung | Howa |
| Trettin | Bettina | Yoo | Dong Whi |
| Trupia | Dilara | Yu | JiaDe |
| Truss | Annie | Zafar | Mubashir |
| Tsai | Wen-Chien | Zamir | Hassan |
| Tschandl | Philipp | Zampella | John |
| Tsou | Christina | Zhai | Kevin |
| Zhang | Hanlin | Zhou | Xinyu |
| zhang | xinyi | ZHU | LELE |
| Zhang | Zewei | Zhuang | Yan |
| Zhao | Shuang | Ziehfreund | Stefanie |
| Zheng | David | Zink | Alexander |
| Zhou | Albert |  |  |
